# Thin-Film-Based SAW Magnetic Field Sensors

**DOI:** 10.3390/s21248166

**Published:** 2021-12-07

**Authors:** Jana Marie Meyer, Viktor Schell, Jingxiang Su, Simon Fichtner, Erdem Yarar, Florian Niekiel, Thorsten Giese, Anne Kittmann, Lars Thormählen, Vadim Lebedev, Stefan Moench, Agnė Žukauskaitė, Eckhard Quandt, Fabian Lofink

**Affiliations:** 1 Fraunhofer Institute for Silicon Technology ISIT, Fraunhoferstrasse 1, 25524 Itzehoe, Germany; jsu@mailbox.org (J.S.); simon.fichtner@isit.fraunhofer.de (S.F.); erdem.yarar@isit.fraunhofer.de (E.Y.); florian.niekiel@isit.fraunhofer.de (F.N.); thorsten.giese@isit.fraunhofer.de (T.G.); fabian.lofink@isit.fraunhofer.de (F.L.); 2 Institute for Materials Science, Kiel University, Kaiserstraße 2, 24143 Kiel, Germany; visc@tf.uni-kiel.de (V.S.); anki@tf.uni-kiel.de (A.K.); lath@tf.uni-kiel.de (L.T.); eq@tf.uni-kiel.de (E.Q.); 3 Fraunhofer Institute for Applied Solid State Physics IAF, Tullastrasse 72, 79108 Freiburg, Germany; vadim.lebedev@iaf.fraunhofer.de (V.L.); stefan.moench@iaf.fraunhofer.de (S.M.); agne.zukauskaite@iaf.fraunhofer.de (A.Ž.)

**Keywords:** surface acoustic waves, surface acoustic wave sensor, magnetic field sensor, current sensor, magnetostriction, AlScN, FeCoSiB, MEMS, thin film

## Abstract

In this work, the first surface acoustic-wave-based magnetic field sensor using thin-film AlScN as piezoelectric material deposited on a silicon substrate is presented. The fabrication is based on standard semiconductor technology. The acoustically active area consists of an AlScN layer that can be excited with interdigital transducers, a smoothing SiO_2_ layer, and a magnetostrictive FeCoSiB film. The detection limit of this sensor is 2.4 nT/$\sqrt{\mathrm{Hz}}$ at 10 Hz and 72 pT/$\sqrt{\mathrm{Hz}}$ at 10 kHz at an input power of 20 dBm. The dynamic range was found to span from about ±1.7 mT to the corresponding limit of detection, leading to an interval of about 8 orders of magnitude. Fabrication, achieved sensitivity, and noise floor of the sensors are presented.

## 1. Introduction

The sensing of magnetic fields has a multitude of use cases ranging from biomedical applications to current sensing in automotive applications [1,2,3,4,5], each having different requirements on the sensor regarding bandwidth, dynamic range, dc capability, size, and price [6,7].

A promising sensing principle of magnetic fields is based on surface acoustic waves

(SAW) [8] and the change of the Young’s modulus (∆*E* effect) of magnetostrictive films [9]. This differs from other sensor approaches, such as using a magnetoelectric composite cantilever suffering from disadvantages such as a small bandwidth, and a good LOD that can only be achieved in resonance [10].

Today, for the fabrication of SAW sensors, the use of piezoelectric single-crystal substrates such as quartz [1,11,12] or LiNbO_3_ [13,14] is state-of-the-art. For ST-cut quartz sensors, sensitivities of up to 2000°/mT and a limit of detection of 100 pT/Hz^1/2^ at 10 Hz are reached [15] and, for LiNbO_3_, a variation of the SAW velocity of Δ*v*/*v* = 0.27% at 400 mT [14]. To enable a greater material flexibility, especially in terms of compatibility with CMOS and MEMS technology, a reduction in chip size and the use of cleverly designed multilayers to enhance device performance requires a change to thin-film technology.

For that purpose, thin-film AlN is a promising piezoelectric material due to its high wave velocity, good mechanical and dielectric properties, high thermal conductivity, and high breakdown voltage [16]. Additionally, a SAW sensor operation up to several GHz can be realized, which can significantly increase the sensitivity in many sensor applications [17]. Further, it was shown that alloying AlN with Sc improves the electromechanical coupling significantly without losing the attractive material properties of AlN [18]. The electromechanical coupling in AlScN even increases with increasing frequency, so that its use is particularly interesting for high SAW frequencies [19,20]. The Sc concentration adds an additional parameter for tuning crucial properties of SAW devices, such as the phase velocity and the electromechanical coupling [20]. AlScN as a promising thin-film material for SAW sensors, as it is described in [21], is studied in this work, and it can be fabricated at reasonable cost with standard semiconductor technology on larger wafer sizes and an easier process integration compared to bulk piezoelectric wafers.

**Figure 1:**
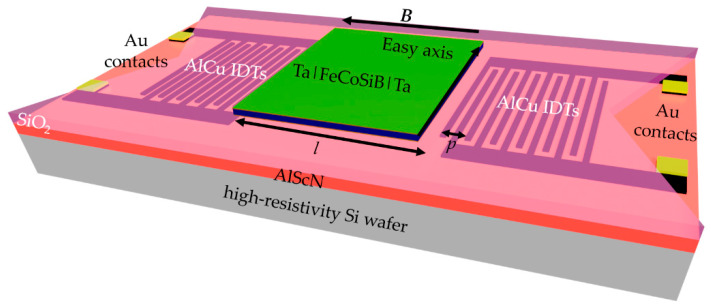
Schematic sketch of the SAW thin-film magnetic field sensor. Magnetostrictive FeCoSiB on top of the silicon dioxide layer of length *l* is in between the AlCu IDTs. FeCoSiB is capped with Ta to avoid corrosion. The easy axis of the magnetostrictive film defines the sensitive direction of the sensor against an external applied magnetic field ***B*** and is chosen to be perpendicular to the direction of SAW propagation. As the piezoelectric material, AlScN is chosen.

For SAW devices, two common design approaches exist: a delay line, and a resonator configuration [22,23]. In this work, the delay line configuration is chosen, as shown in Figure 1, to increase the interaction volume between the excited wave and the magnetic field sensitive area (magnetostrictive film). For this purpose, two inter-digital transducers (IDTs) are structured on the acoustic layer (thin film AlScN) to excite and readout the SAW signal via the piezoelectric effect [24]. The delay line of length *l* is located between the two IDTs. To prevent a short circuit between the magnetostrictive film and the IDTs and, more importantly, to reduce the roughness of the underlaying layer of the magnetostrictive film, a SiO_2_ layer is grown on top of the piezoelectric layer. The topmost layer in this area of the delay line is the magnetostrictive material FeCoSiB. The acoustic wave passing through the delay line couples to an external magnetic field via the induced change in the Young’s modulus of the magnetostrictive film [9].

As the change of Young’s modulus ΔE alters the phase velocity ***ν*** of the acoustic wave [25], a ***B***-field-induced phase change Δφ=2π l f/(*v*(*B*_1_) − *v*(*B*_0_)) can be detected at the output IDTs via the direct piezoelectric effect [26]. The sensitivity of the sensor, which is defined as the phase change per change in magnetic field S=∂φ/∂H, can be written as the product of its individual contributions: magnetic layer sensitivity *S_mag_* (change in Young’s modulus with magnetic field), structural sensitivity *S_str_* (change of the wave velocity with change in Young’s modulus), and geometric sensitivity *S_geo_* (phase change with change in wave velocity) [1]:

(1) $$S=\frac{\partial \varphi }{\partial H}=\frac{\partial G}{\partial H}\cdot \frac{\partial \nu }{\partial G}\cdot \;\frac{\partial \varphi }{\partial \nu }=S_{mag}\cdot \;S_{str}\cdot S_{geo}$$

By means of *S* and the power spectral density $S_{\varphi }$ of the random phase fluctuations of the sensor, the limit of detection (LOD) of the sensor can be calculated by [27]:

(2) $$\mathrm{LOD}=\frac{\sqrt{S_{\varphi }\;}}{S}$$

The logarithmic presentation of the power spectral density 10 log_10_ ($S_{\varphi }$) is referred to as phase noise.

## 2. Materials and Methods

### 2.1. Sensor Fabrication

On a 200 mm, 725 µm thick, single-side polished high-resistivity Si (001) wafer, a 1 µm Al_0.77_Sc_0.23_N layer is sputtered as described in [28]. 

**Figure 2:**
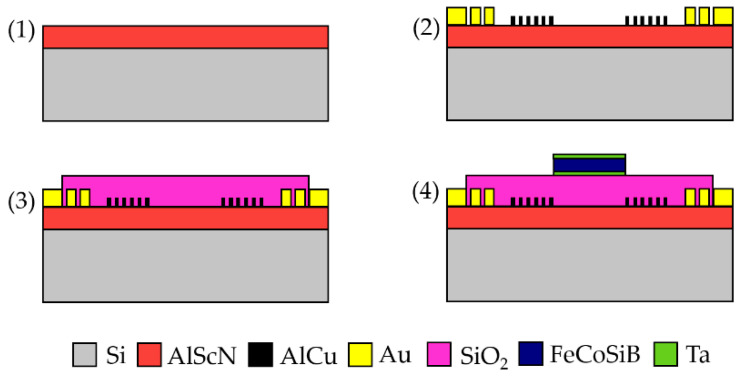
Schematic cross-sections of the processing steps of the thin film SAW sensor. (**1**) A layer of 1 µm AlScN is sputter-deposited on top of a high-resistance silicon (001) wafer, followed by 200 nm AlCu IDTs and 300 nm gold contacts with a 40 nm WTi adhesion layer that are patterned afterwards (**2**). A 1.5 µm SiO_2_ layer is deposited via PECVD and thinned with CMP to a thickness of 1 µm (**3**). On top, the magnetostrictive FeCoSiB film with a thickness of 200 nm is deposited with an additional layer of 10 nm Ta on the top and on the bottom (**4**).

Afterwards, 200 nm thick AlCu IDTs are sputtered and patterned by dry chloride etching to a design with a delay line length of *l* = 3.8 mm, a split-finger structure [29] of 25 pairs, a periodicity of *p* = 16 µm, and a finger width of 2 µm, resulting in a theoretical phase velocity of the Rayleigh-like mode of 283 MHz (Figure 2(1)). Three-hundred-nanometer-thick gold contacts with a 40 nm WTi adhesion layer are sputter-deposited and structured with a wet etching step (Figure 2(2)). A 1.5 µm thick, low-stress SiO_2_ interlayer is deposited with plasma-enhanced chemical vapor deposition (PECVD) at 400 °C and smoothed and thinned with a chemical mechanical polishing (CMP) step to a thickness of 1 µm. An atomic force microscopy analysis showed that this reduces the surface roughness from 2 nm of the AlScN layer to a roughness of below 1 nm. Such a reduction significantly enhances the soft magnetic properties of the FeCoSiB thin film [30]. Afterwards, the layer is structured with dry etching (Figure 2(3)). 

**Figure 3:**
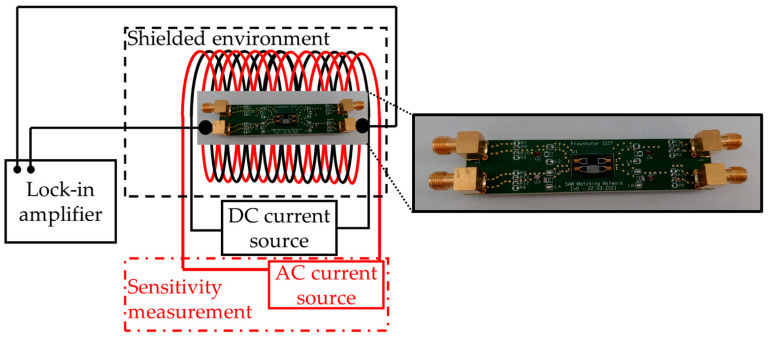
Sketch of the measurement setup. The SAW sensor is placed in a magnetically, electrically, and acoustically shielded measurement chamber inside of two solenoids. For the phase shift measurements, only a dc current source is used to apply a homogeneous magnetic field. A lock-in amplifier is used to apply the synchronous SAW frequency and measure the phase change. The sensitivity *S* is measured by applying an additional ac magnetic field using the second solenoid that is supplied with another current source using a test amplitude and frequency. The inset shows a zoom-in of the ready-to-use sensor with a balun attached to symmetrize the signal.

The magnetostrictive layer consisting of 200 nm (Fe_90_Co_10_)_78_Si_12_B_10_ is deposited via RF magnetron sputtering on top of the SiO_2_ layer and structured with ion beam etching to realize steep and straight edges. To improve adhesion and prevent oxidation, 10 nm Ta is deposited on top and below the FeCoSiB film (Figure 2(4)). To induce a uniaxial magnetic anisotropy in the soft magnetic film, an annealing step at 250 °C for 30 min is performed while applying a magnetic field of 0.2 T. Thereby, the easy axis of the FeCoSiB film is aligned perpendicular to the SAW propagation direction (see Figure 1). The simple process of thermal alignment of the magnetization is an example of the integration-related advantages of the silicon substrate-based thin-film concept. When using single crystal piezoelectric substrates, such a simple thermal imprint is not possible due to anisotropic thermal expansion in the piezoelectric substrate and would result in a significant reduction of the soft magnetic film properties. Instead, a more complex, low-temperature deposition with an applied magnetic field must be applied to achieve a proper alignment of the magnetization [26]. Finally, the sensor is glued and wire-bonded on top of a printed circuit board (PCB), on which there are balun devices to symmetrize the signal. The final sensor is shown in the inset of Figure 3.

### 2.2. Experimental Setup

The sensor’s two-port scattering parameters (S-parameters) are characterized with a vector network analyzer E8361A from Agilent Technologies. A signal power of *p* = 0 dBm is used throughout the experiments in this paper, except for the noise measurements. For all measurements in a magnetic field, the sensor is placed in the center of two axially stacked coils, which are used to generate ac and dc magnetic signals by means of a programmable current source (KEPCO BOP20-10ML) for the dc magnetic field. The solenoids are placed inside a magnetically, electrically, and acoustically shielded measurement chamber. The magnetic field shielding is provided by a mu-metal cylinder ZG1 from Aaronia AG to prevent external influences. The magnetically induced phase shift of the sensor is measured in the homogeneous magnetic field region of the solenoids. To then record the sensor behavior in the magnetic field, the magnetic flux density is swept from negative to positive values and reversed. A lock-in amplifier (UHFLI from Zurich Instruments) is used to apply the synchronous SAW frequency determined by the measurement of the S-parameters and to measure the static phase response $\varphi \left(B\right)$ of the sensor at a chosen input power (here 0 dBm).

In order to determine the sensor’s optimum working point with the highest sensitivity, the phase φ is analyzed as a function of a dc bias field H. In principle, a numerical calculation of the sensitivity S=∂φ/∂H should be sufficient to determine the point of steepest slope, which refers to the point of highest sensitivity, but often small phase jumps related to domain wall movement can give the appearance of incorrectly high sensitivities. Therefore, a dynamic phase detection measurement is performed to accurately determine ∂φ/∂H at every single measurement point. For this, one solenoid generates the static magnetic bias field that is superimposed with an ac test-field generated with the second solenoid powered by a current source (Keithley 6221) with a defined amplitude of 10 µT and a frequency of 10 Hz. By choosing the amplitude of the ac field that is large enough, the phase fluctuations can be neglected. The sensor’s output signal and the phase reference are fed into the UHFLI lock-in amplifier that is used as a phase demodulator. The phase sensitivity is obtained by the evaluation of the amplitude spectrum of the demodulated phase signal [26]. 

The phase noise measurements are performed with the Rohde & Schwarz FSWP phase noise analyzer at the sensor’s magnetic working point and at magnetic saturation at a different sensor’s input power. The SAW sensor is placed in the electrically, magnetically, and acoustically shielded chamber during these measurements. To minimize external noise sources, especially those appearing in common dc current sources, a battery-based current source controlled by a potentiometer is applied in series with the solenoids for the generation of the dc magnetic bias field. The internal generator of the phase noise analyzer excites the sensor at the synchronous SAW frequency determined in previous measurements. The LOD can be determined from the measured noise floor with Equation (2). A more detailed description of the measurement setup can be found in [27].

## 3. Sensor Characterization

**Figure 4:**
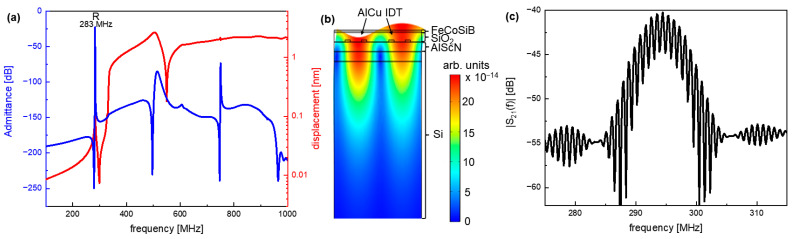
(**a**) FEM-simulated displacement (red) and admittance (blue) for the presented sensor design. (**b**) Colored map of absolute deflection for the Rayleigh-like mode at 283 MHz. The deflection into the FeCoSiB layer, the SiO_2_ intermediate layer, the IDTs, the AlScN layer, and the Si substrate are displayed. (**c**) Measured transmission behavior (scattering parameter S_21_) of the presented sensor. The synchronous frequency of the sensor is determined to be 294.2 MHz with a return loss of 40 dB.

A finite element method (FEM) analysis and spectra analysis were performed using COMSOL Multiphysics^®^ software [31] based on the acoustic and electromagnetic parameters of the constituent layers (AlScN, SiO_2_, FeCoSiB). The parameters for AlScN were taken from [21] and for FeCoSiB from [1]. The simulated admittance and the displacement of the SAW modes are shown in Figure 4a. A Rayleigh-like thin-film mode is simulated to be at 283 MHz with a high admittance and relatively high displacement that are defined on the surface with some energy losses in the direction of the Si substrate (see Figure 4b).

The measured transmission behavior of the thin-film magnetic field sensor is shown in Figure 4c, exhibiting a synchronous frequency of 294.2 MHz at zero flux density, which is very close to the simulated value. The deviation can be explained by the material parameters in the simulation deviating from the experimental parameters in the real sensor, or imperfections in the fabrication, and is assessed as low.

**Figure 5:**
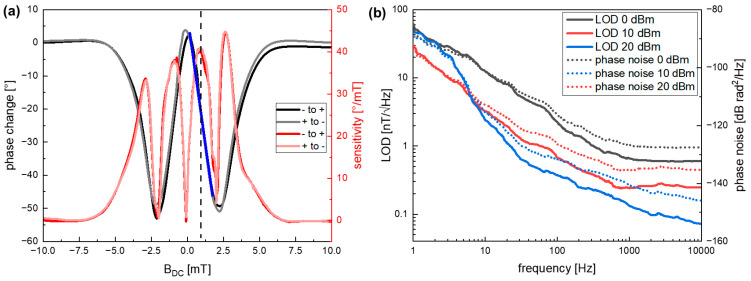
(**a**) The induced phase shift in the sensor with an external magnetic flux density (black) and the direct measurement of the sensitivity with an ac test signal (red) is shown. The highest slope in the phase change occurs at about 0.85 mT and 2.65 mT, resulting in the highest values of sensitivity of up to 45°/mT. A value of 0.85 mT is chosen as a working point (indicated with the dotted line) due to the high sensitivity and the lower field value compared to 2.65 mT. The dynamic range of the sensor is marked with the blue line, showing the linear region of the sensor. The ac signal has an amplitude of 10 µT and a frequency of 10 Hz. (**b**) Measured phase noise (dotted line) and calculated limit of detection (solid line) as a function of the frequency at magnetic saturation for 0 dBm (black), 10 dBm (red), and 20 dBm (blue) input power.

The performance of the sensor in a magnetic field is measured as described above by applying the synchronous frequency of 294.2 MHz with the lock-in amplifier and measuring the static phase response of the sensor shown in Figure 5a from negative to positive field values (black) and reverse (grey). A slight hysteresis is observable, as was expected, resulting from the magnetic material. The linear region of the static phase response, which determines the dynamic range, is marked with a blue line in Figure 5a.

The sensitivity that is given by the derivative of the phase change as described above is important for the performance of the sensor. The dynamically measured sensitivity via an ac test signal depending on the bias field is shown in Figure 5a (red). The two regions with the maximum phase change are observed at about 0.85 mT and 2.65 mT (Figure 5a) and are the most interesting for sensor application. The highest sensitivity of about 45°/mT is reached at 2.65 mT.

Besides a high sensitivity, a good LOD is an important sensor parameter of the SAW sensor [15]. The LOD dependency on frequency from the carrier and sensor input power is shown in Figure 5b. The measurements are performed at the sensor’s working point at *H_bias_* = 0.85 mT, which is chosen due to the technological limitation of the LOD measurement setup and at magnetic saturation. As both measurements are almost identical, only the measurement at saturation is shown in Figure 5b.

Up to a frequency of 1 kHz, a regime of flicker (1/f) noise dominates the spectra. This noise is related to defects in the substrate and the SiO_2_ layer, as well as random fluctuations of the magnetization and magnetic hysteresis losses [27]. In the specific case of SiO_2_, additional surface roughness is introduced during the ion beam etching step to pattern the FeCoSiB layer. A possible way to reduce this roughness would be to add a lift-off process, though this would have the disadvantage of less defined edges of the magnetostrictive film.

In the 1/f noise regime, a LOD of 3.2 nT/Hz^1/2^ is achieved at 10 Hz and an input power of 10 dBm. Above 1 kHz, the noise is dominated by white noise, which is additive noise and decreases with increasing signal power [27]. Here, a LOD of 246 pT/Hz^1/2^ is reached for 10 kHz. When the input power is increased to 20 dBm, the LOD can be decreased even further at higher frequencies above 10 Hz so that a LOD of 2.4 nT/Hz^1/2^ can be reached at 10 Hz and 72 pT/Hz^1/2^ at 10 kHz. 

The dynamic range of the sensor is given by the linear region around the working point of the sensor and is indicated in Figure 5a with the blue line. It spans from about 1.7 mT to the corresponding LOD, leading to an interval of 8 orders of magnitude. The hysteretic behavior could be compensated as is done in AMR sensors (anisotropic magnetoresistance) with controlled current pulses [32].

With these characteristics, our sensor has already high potential for sensing a wide range of technically relevant electrical currents via the generated magnetic field. In contrast, other current sensor concepts, such as Hall sensors, are limited in the bandwidth in the needed dynamic range and cannot achieve the measurement of fast signals [33]. AMR sensors also have a limit of the bandwidth at 1 MHz and a low dynamic range [34]. The presented SAW sensor is dc-compatible with a moderately high bandwidth of about 1.2 MHz limited by the delay line length and a high dynamic range of about 8 orders of magnitude.

## 4. Conclusions

The first thin-film SAW magnetic field sensor using AlScN as piezoelectric material on a silicon substrate is presented. The limit of detection is 2.4 nT/Hz^1/2^ at 10 Hz, which probably can be lowered further with an impedance matching, higher input power values, and further insights on the sensor design and noise sources. This will also have a high impact on the phase change and the resulting sensitivity of the sensors. At higher frequencies above 10 kHz, the LOD is found to be as low as 72 pT/Hz^1/2^. Additionally, the magnetic layer can be optimized by an exchange bias [35] to eliminate the need for an external bias field. Due to the possibility to measure galvanically isolated values from dc up to MHz with a high dynamic of up to 8 orders of magnitude, the presented sensor is very interesting for a variety of modern measuring tasks, such as the control of modern power switches, where it is well suited for monolithic wafer-level integration circuits. This clearly sets it apart from the competition in the segment of galvanically isolated magnetic field sensors for power transformers in the field of electromobility, which include Hall sensors and AMR sensors. Thus, this sensor concept has the potential to manage the rising requirements on current sensors regarding bandwidth, dynamic range, precision, and compactness.

## Data Availability

The data presented in this study are available on reasonable request from the corresponding author.
